# Neuroinflammation in Autism and Supplementation Based on Omega-3 Polyunsaturated Fatty Acids: A Narrative Review

**DOI:** 10.3390/medicina57090893

**Published:** 2021-08-28

**Authors:** Aleksandra Veselinović, Snježana Petrović, Vladica Žikić, Miško Subotić, Vladimir Jakovljević, Nevena Jeremić, Vesna Vučić

**Affiliations:** 1 Cognitive Neuroscience Department, Research and Development Institute “Life Activities Advancement Centre”, 11000 Belgrade, Serbia; v.zikic@iefpg.org.rs (V.Ž.); m.subotic@add-for-life.com (M.S.); 2 Department of Speech, Language and Hearing Sciences, Institute for Experimental Phonetics and Speech Pathology, 11000 Belgrade, Serbia; 3 Group for Nutritional Biochemistry and Dietology, Centre of Research Excellence in Nutrition and Metabolism, Institute for Medical Research, National Institute of Republic of Serbia, 11000 Belgrade, Serbia; snjezana.petrovic@imi.bg.ac.rs (S.P.); vesna.vucic@imi.bg.ac.rs (V.V.); 4 Department of Physiology, Faculty of Medical Sciences, University of Kragujevac, 34000 Kragujevac, Serbia; drvladakgbg@yahoo.com; 5 Department of Human Pathology, 1st Moscow State Medical University IM Sechenov, 119991 Moscow, Russia; 6 Department of Pharmacy, Faculty of Medical Sciences, University of Kragujevac, 34000 Kragujevac, Serbia; n.barudzic@hotmail.com

**Keywords:** ASD, PUFA, supplementation, neuroinflammation, gut microbiota, gut–brain axis

## Abstract

Autism Spectrum Disorder (ASD) is a complex neurodevelopmental disorder characterized by persistent deficits in social communication and social interaction across multiple contexts and restricted, repetitive patterns of behavior, interests and activities. The maternal status of polyunsaturated fatty acids (PUFA) regulates microglial activity and neuroinflammatory pathways during a child’s brain development. In children with ASD, the metabolism of PUFA is thought to be deficient or abnormal, leading to increased production of proinflammatory cytokines, increased oxidative stress and an imbalance in the formation and action of neurotransmitters. In addition, nutritional deficits in omega-3 PUFA may affect gut microbiota and contribute to ASD by the gut–brain axis. The aim of this study was to review the possible role of neuroinflammation in ASD development and the effect of omega-3 PUFA supplementation in children with ASD. Due to a wide heterogeneity across RCTs, no definitive conclusion about omega-3 PUFA effects in ASD can be drawn. Supplementation with PUFA could be considered as one of the aspects in regulating the biological status of the organism and could provide added value to standard medical and psychological interventions for reducing behavioral deficits.

## 1. Introduction

Autism spectrum disorder (ASD) is defined by the American Psychiatric Association as a complex neurodevelopmental disorder characterized by persistent deficits in social communication and social interaction across multiple contexts and restricted, repetitive patterns of behavior, interests and activities, according to the Diagnostic and Statistical Manual for Mental Health, Fifth Edition (DSM-5) [1]. These symptoms must be present in the early developmental period and produce clinically significant developmental impairment in social, occupational or other important areas of current functioning [1,2,3].

The exact cause of ASD is unclear, but a combination of different risk factors and genetic and environmental factors is assumed [4]. The conventional perspective of ASD symptoms suggests that ASD is a genetic disorder that involves a complex genetic background [3,5]. Several research directions support the belief that a combination of complex neurobiological, environmental, immunological and genetic factors is crucial in the etiology of autism. Additionally, proinflammatory cytokines secreted by several types of different cells contribute to the pathogenesis of neuroinflammation [6].

Many patients with ASD have associated medical conditions such as anxiety, sleep disorders, metabolic disorders, eating disorders and gastrointestinal (GI) problems that have a significant impact on the quality of patients and their caregiver’s lives [7]. Studies of immune dysregulations and GI problems within the neuroimmune system of patients with ASD are particularly interesting to many scientists [8].

The aim of this study was to review the possible role of neuroinflammation in the development and progression of ASD and the effects of omega-3 PUFA supplementation in children with ASD. Given that a wide range of symptoms is related to ASD, the issues about the broad biological background of autistic symptoms and the potential beneficial effect of omega-3 PUFA supplementation are discussed.

## 2. Gut–Brain Axis (GBA) and ASD

The human gut contains up to 100 trillion microorganisms, including at least 1000 different types of (so far) known bacteria, that collectively affect the host’s digestive, immune, metabolic and nervous systems [9,10]. The gut microbiome (microbiota) is mainly formed during the first months of life under the influence of various factors such as vaginal birth, host genome, formula feeding, antibiotic use, GI infections and stress [9,10,11].

**Figure 1:**
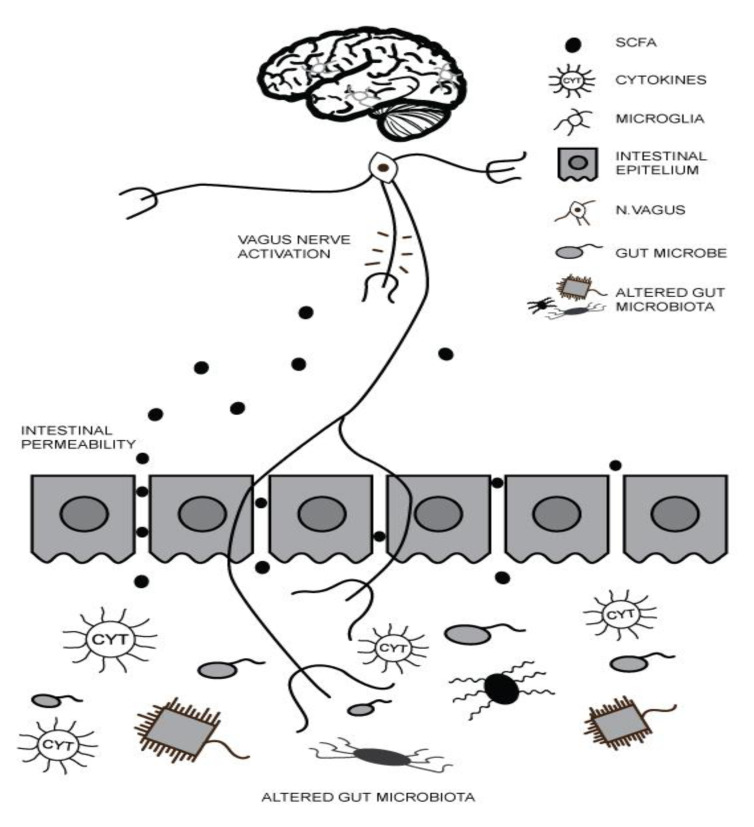
Altered gut microbiota and inflammatory processes in Autism Spectrum Disorder (ASD).

A growing body of evidence supports the hypothesis that gut microbiota plays a vital role in neuroinflammation [12]. Communication along the microbiota–GBA mainly describes how signals from the gut microbiota influence brain function, as well as how brain messages impact microbiota activity and GI physiology [13]. Disturbances within the microbiota–GBA have been suggested as potential contributors to the occurrence and development of ASD. The balance of inflammatory cytokines is skewed, and intestinal permeability seems to be increased in children with ASD who display gastrointestinal symptomatology when compared to those children who did not [14]. Short-chain fatty acids (SCFA) are critical mediators in creating a link between the gut microbiota and the brain, as they cross the blood–brain barrier and directly affect changes in brain activity [13,15]. The three types of SCFA are acetic acid, valeric acid and propionic acid [16], and these SCFAs are important for the health and regulation of the small intestine membrane and the development of native and adaptive immune responses [10,11,17]. The pathway of the initial immune response is evoked by the production of bacterial toxins (e.g., *Clostridia* spp.) by the gut microbiota, which further evokes the immune response in the gut as well as in the bloodstream; and increased oxidative stress occurs in this type of immune response. Afterwards, oxidative stress on epithelial membranes increases intestinal permeability, resulting in bacterial translocation into lamina propria of mesenteric lymphoid tissue. Subsequently, mucosal immune cells, macrophages and dendritic cells release proinflammatory cytokines [10,11,18]. Proinflammatory cytokines then activate the vagus nerve or reach the brain (Figure 1) through the bloodstream, and in this way, they regulate the activity of microglia and the functioning of the Central Nervous System (CNS) [10,11,18]. Microglial cells are a type of macrophages that act as the first defense mechanism of the brain immune system, and these unique, resident, immune cells of the CNS monitor the CNS and synaptic discharges during normal neural development and represent the primary mediators of inflammation [6].

The gut microbiota exhibits important bidirectional interactions with the immune system. Many facets of immunity are dysregulated in ASD [8]. Neuroinflammation affects the composition of the microbiota and vice versa, and nutritional deficits in omega-3 fatty acids can be an important risk factor for ASD [17].

## 3. Maternal Inflammation during Pregnancy and ASD

Epidemiological studies indicate a strong association between maternal inflammation and the pathogenesis of ASD [17,18]. During pregnancy, pathogens are thought to increase the risk of neurodevelopmental disorders in the offspring depending on the timing of infection and the magnitude of the maternal immune response [17]. Pathogenic microbiota, bacterial metabolites and their components can stimulate the secretion of proinflammatory cytokines [9,19]. These maternal cytokines can cross the placental barrier and stimulate de novo synthesis of cytokines in the fetal brain, which makes the fetal brain sensitive to neurodevelopmental changes [17]. Additionally, placenta inflammation can induce a systemic fetal inflammatory response that contributes to white matter damage in the fetal brain. This type of inflammation also leads to neonatal brain damage [19]. The presence of various levels of inflammatory cytokines has been found in blood, especially in monocytes, serum and plasma, as well as in the brain tissue and cerebrospinal fluid of patients with ASD, which leads to impairment in CNS immune capacity and enhanced activation of microglia in the brain [6,20]. Chronic microglial activation contributes to the development and progression of neurodegenerative disorders. Active microglia can induce the production of proinflammatory cytokines such as interleukin 1 β (IL-1 β), interleukin-6 (IL-6) and tumor necrosis factor α (TNF-α), which are typically intended to prevent further damage of the brain tissue. Abnormal microglia activation is sometimes toxic to neurons and other glial cells [6].

Many studies have confirmed the presence of activated microglia, accompanied by proinflammatory factors such as cytokines and chemokines in the brain and in cerebrospinal fluid in the dorsolateral prefrontal cortex of patients with ASD. Deficits in microglial activity during brain development lead to an increased number of immature synapses, and thus to the cognitive impairments and behavioral disorders typical for patients with ASD [17]. Inflammation that occurs in the form of elevated cytokine levels transmit across the blood–brain barrier and initiate a neuroinflammatory response that has been offered as an explanation for later neurodevelopmental complications, including cerebral palsy, autism, schizophrenia and cognitive impairments [19]. Although supplementation with PUFA can reduce inflammation and improve the balance between pro- and anti-inflammatory cytokines, the effects of PUFA on later neurodevelopmental complications are not clear.

The immunophenotypes that can be seen in patients with ASD are characterized by elevated proinflammatory status, i.e., elevated levels of cytokines and chemokines, including IL-1β, IL-6, interferon µ (IFN-µ), tumor necrosis factor α (TNF-α), subunit beta of interleukin 12 (also known as IL-12 subunit p40), MCP-1 (monocyte chemoattractant protein 1, cytokine also known as CCL-2), TGF-β (transforming growth factor-β) as well as hyperactive cellular immune responses [21]. However, immune abnormalities, including differentiating the immune/cytokine profile, are the result of the diet, lifestyle and genetic profile of each patient with ASD [8], and this makes it difficult to pinpoint the links between maternal inflammation, diet/supplementation and ASD changes in children [22].

## 4. Essential Fatty Acids—Omega-3 and Omega-6 PUFA and Inflammatory Processes in ASD

PUFA are important constituents of phospholipids, which play an essential role in cell membrane structure and function [23]. There are crucial structural and functional components of cellular and intracellular membranes in the human body, and those are linoleic acid (18:2*n*-6, LA), α-linolenic acid (18:3*n*-3, ALA) and their metabolic products, arachidonic acid (20:4*n*-6, AA), eicosapentaenoic acid (20:5*n*-3; EPA) and docosahexaenoic acid (22:6*n*-3; DHA).

ALA, the precursor of omega-3 PUFA, can be converted into EPA and further to docosapentaenoic acid (22:5*n*-3; DPA) and DHA. DHA plays a role in cognitive functions and in neurite growth, membrane fluidity, neurotransmission, endothelial function, neuronal survival and attenuating neurodegeneration [24]. Therefore, its intake is very important during pregnancy and in young children. AA is the precursor of proinflammatory eicosanoids, including prostaglandins [23,25], which contribute to the occurrence of allergies and inflammatory disorders that are very frequently associated with ASD [26,27]. Eicosanoids derived from omega-6 AA have opposing properties from those originating from omega-3 EPA. Up to 60% of patients with ASD have some systemic immune dysfunction, indicating a link between PUFA and inflammatory homeostasis in ASD [28], which is associated with impaired omega-6/omega-3 PUFA in the diet. Blood omega-3 levels of long-chain PUFA (LCPUFA) are reduced in children with ASD [29], which can lead to hyperproduction of proinflammatory cytokines derived from omega-6 [30]. Additionally, elevated levels of autoantibodies to neuronal and glial molecules in patients with ASD can be attributed to disorders of the omega-6/omega-3 PUFA ratio [31].

Lower levels of omega-3 PUFA EPA and DHA and higher AA/EPA ratio were found in children with ADHD and ASD compared with typically developing controls in the study of Parletta et al. (2016), and these levels were correlated with greater severity of symptoms. Attention seems to be an essential characteristic of neurodevelopmental disorders, especially in learning disorders [24]. Altered PUFA metabolism leads to increased production of proinflammatory cytokines, increased oxidative stress and an imbalance in the formation and action of neurotransmitters in individuals with ASD [32]. Children with increased inflammation may benefit more from daily vitamin D and omega-3 LCPUFA supplements because of the modulated responses to their inflammatory state as a part of the supplementation [33]. The structural and functional roles of PUFA in neuronal membranes support underlying biological mechanisms and suggest that supplementation can help with symptoms in children with psychiatric disorders [24].

### 4.1. Omega-3 and Omega-6 PUFA and Gut Microbiota in ASD

The exact mechanism of the PUFA effects in ASD is not quite clear, but one possibility is that PUFA affects the brain through GBA. Understanding gut–brain interactions may open novel approaches to treat mood and behavioral disorders [34]. A synergetic effect between omega-3 PUFA and probiotic bacteria during digestion has been recorded, where omega-3 lipids promoted the probiotic attachment to the intestinal wall [35]. Thus, microbiota-related treatment strategy can find support in studies where mice fed with the omega-3-supplemented diet displayed greater fecal *Bifidobacterium* and *Lactobacillus* abundance with healthy hypothalamus–pituitary–adrenal axis activity under stressful conditions. These results show that dietary intervention, in particular with omega-3 PUFA, may have an impact on behavioral outcomes, and it could be mediated by the GBA [34,36].

Higher omega-6 PUFA intake has been associated with decreased numbers of *Bifidobacteria* and in a decrease in certain immune functions, such as expression of antigens and proinflammatory cytokines [37,38]. This is important because *Bifidobacteria* improve intestinal epithelial integrity, reduce inflammation and have a positive effect on anxiety and behavioral disorders in rodents [36]. Additionally, intake of omega-3 PUFA has been shown to have a significant positive association with *Lactobacillus* group abundance in feces samples. The increase in omega-3 PUFA is effective in supporting epithelial barrier integrity by improving transepithelial resistance and by reducing IL-4-mediated permeability, and several *Lactobacilli* enhance the function of the intestinal barrier [38]. These results suggest a link between PUFA types and their different effects on the fecal microbiota. As a result, balanced fat intake is crucial for the creation of the microbiota and overall for the health of the host [37].

It is indicated that essential fatty acids have a beneficial role in postnatal interventions; thus, metabolic interaction between omega-3 PUFA and intestinal bacteria should be identified and noticed as effective to the immunity and enteric nervous system and further for improvement of behavior.

Another explanation of neurodevelopmental disorders could involve the influence of gut microbiota on PUFA uptake and metabolism; thus, the other direction of omega-3 PUFA and probiotics interaction should not be ignored. Dietary supplementation with Bifidobacteria has been shown to increase tissue levels of EPA and DHA in mice, and dietary supplementation with parent omega-3 PUFA ALA combined with *Bifidobacterium breve* resulted in higher liver EPA and brain DHA levels [39]. Gut endothelial barrier integrity may also be enhanced by the PUFA dihomo g-linolenic acid (20:3*n*-6, DGLA), AA, EPA and DHA [40]. It has been observed that probiotic bacteria (e.g., *Bifidobacterium* and *Akkermansia*) and omega-3 fatty acids may have synergistic health benefits in treating patients with ASD. Decreased levels of *Akkermansia*, which is a bacterial species that lives inside the mucus layer of the gut and supports the intestinal wall [41], may lead to a weakened barrier function and increased gut permeability as an autistic phenotype. Rodríguez-Carrio et al. [42] noted an opposite pattern between the *Akkermansia* and *Lactobacillus* groups, with *Lactobacillus* being overgrown in patients with ASD who have impaired FA metabolism [34]. Decreased *Akkermansia* levels were associated with elevated serum interleukin IL -6 and impaired free fatty acid profile.

Additionally, the effect of omega-3 during pregnancy should be considered, which would potentially prevent the consequences of neuro-developmental impairment.

### 4.2. Maternal Status of PUFA during Pregnancy and ASD

The maternal status of omega-3 PUFA has been shown not only to regulate microglial activity and neuroinflammatory pathways during brain development but also to affect brain plasticity [17]. The intake of omega-3 PUFA in pregnancy or in the early postnatal period and changes in the structure of gut microbiota can influence the occurrence of neuropsychiatric disorders [36]. These authors highlighted that in utero and early life, omega-3 PUFA intake, particularly EPA and DHA, regulates gut microbiota development, influencing the bacterial abundance and types in adolescence and adulthood and affects social and communicative behavior throughout one’s lifespan [43]. Reduced early life omega-3 intake can induce discrete psychiatric abnormalities in the adolescence period that manifest as communication, behavior and memory disorders. Omega-3 PUFA is essential for preventing behavioral and neurodegenerative disorders later in life [36,44]. A deficit of DHA during the perinatal period was shown to be associated with a reduction in neurogenesis and delays in neuronal migration [45]. These results suggest that the availability of adequate amounts of various PUFA (both omega-6 and omega-3 fatty acids), especially AA and DHA, to the fetus and newborn is recommended [35,46]. In fact, women with a higher intake of PUFA before and during pregnancy [47] had a reduced risk of having a child with ASD than those with lower PUFA intake [43,48]. Omega-3 PUFA is recommended for consumption during the last week of gestation and the first month postnatal for brain development; supplementation with omega-3 PUFA, especially during pregnancy and lactation, may help prevent ASD in children at risk [45].

Steenweg-de Graaff et al. [47] further indicated that more than a deficit of DHA intake, a higher maternal omega-6 to omega-3 PUFA ratio during pregnancy was associated not only with worse neurodevelopmental outcomes but also with a higher number of autism traits in offspring [47], and these findings suggest that the focus of dietary interventions should not be only the increasing of omega-3 PUFA intake but also the reduction of food intake with a high content of omega-6 PUFA [45].

In the prospective study of high-risk younger sibling pregnancies, an association between higher maternal total omega-3 PUFA intake in the second half of pregnancy and reduced risk of ASD in the offspring was evaluated. The maternal third trimester of plasma PUFA concentration was measured by gas chromatography. In all, 258 mother-child pairs were included. Mothers consuming more total omega-3 in the second half of pregnancy were 40% less likely to have children with ASD. This study provides suggestive evidence of associations between risk of ASD in the children and maternal omega-3 intake in late pregnancy but not with third-trimester plasma PUFA [49].

## 5. The Role of Omega-3 PUFA Supplementation in ASD Symptoms

A limited number of RCTs investigated the effect of omega-3 PUFA supplementation on autism symptoms as well as on the improvement of behavioral issues (Table 1). Among those, 12 high-quality RCTs will be discussed in detail since they: (1) have the diagnosis of ASD according to the DSM criteria; (2) include children from 18 months old, adolescents and adults up to 28 years old; (3) are RCTs with an adequate protocol (placebo or no intervention); (4) investigate how omega-3 PUFA intake affects autistic symptoms.

Studies on the effects of omega-3 PUFA on ASD differed in types of PUFA (EPA, DHA or both), doses, duration and if they used a combination of omega-3 PUFA with vitamins or omega-6 and omega-9 PUFA. Table 1 summarizes the key characteristics of the RCTs investigating the effect of omega-3 PUFA supplementation on ASD. Several RCTs results showed that omega-3 PUFA supplementation improved some core symptoms of ASD, in particular hyperactivity [50,51], lethargy [50,52] and stereotypy [50,51,52] (Table 1). Bent et al. [51] treated children with ASD who had hyperactivity for 6 weeks with daily doses of 1.3 g of omega-3 fatty acids compared to placebo. Some improvement in the hyperactivity scores of the omega-3-treated children was observed with a non-statistically significant difference; some statistically greater improvements in the stereotypy and lethargy subscales were found. Yui et al. [52] treated seven children with ASD with large doses of AA and DHA for 16 weeks and compared them to six children who received a placebo. They observed a significant improvement in the social withdrawal subscale, in stereotyped and repetitive behavior and in the communication subscale.

Voigt et al. [53] treated children with ASD who were between 3 and 10 years old with 200 mg/day of DHA for 6 months but did not find any improvement in core symptoms of autism. The authors only found a favorable change in functional communication reported by teachers in children with autism who received DHA supplementation. These findings reported a small but not significant benefit of omega-3 PUFA supplementation in children with ASD [54]. Another 6-month RCT of omega-3 fatty acid supplements (1.5 g) vs. placebo in children 2–5 years of age with ASD does not support high-dose supplementation of omega-3 fatty acids in young children with ASD [55].

Meguid et al. [56] found that supplements of DHA, EPA and AA for 3 weeks led to improved behavior in two-thirds of children with ASD, and the authors concluded that PUFA supplementation might play an important role in ameliorating the autistic features and improving their concentration ability, eye contact, motor skills and language development. A possible beneficial effect of the association of omega-3 and omega-6 PUFA in language development was also found in one study performed in preterm children exhibiting ASD [30]. Language and speech issues are key components of ASD symptoms current therapies find difficult to face. In the randomized, double-blind, placebo-controlled trial, Sheppard et al. [30] demonstrated that 3 months of oral treatment with omega-3/-6/-9 fatty acids were able to increase the number of words produced, the combined gesture and word use, and the broader social-communicative gesture in 18–38 months, preterm born, ASD toddlers. They concluded that supplementation with PUFA positively affected overall social communication.

Additionally, Parellada et al. [57] found that social motivation improves during the trial, and the authors concluded that all groups benefit from PUFA supplementation, but patients with lower baseline omega 3/omega 6 show a larger effect. Raine et al. [58] are considering some support for omega-3 as a supplement to standard interventions for clinic-referred children with reactive aggression. Much more limited support was found for the efficacy of omega-3 for antisocial behavior. An RCT conducted by Mazahery et al. [33] demonstrated the efficacy of combined treatment with vitamin D and omega-3 PUFA in increasing social communicative functions in children with ASD. Another trial from the same author reported a significant improvement in symptoms of irritability and in social domains (social awareness and social-communicative functioning) [59]. Keim et al. [60] found clinically significant improvements in ASD symptoms for children 18–38 months of age who were born at ≤29 weeks of gestation randomly assigned to receive Omega-3-6-9 Junior, but effects were confined to one subscale.

To our knowledge, the latest research in this area was performed by Doaei et al. [61], finding that omega-3 treatment improved autism characteristics in children, including stereotyped behaviors and social communication, and the GARS score after the intervention compared to the control group. The authors found no significant change in the score of social interaction subscale.

Based on conducted studies, omega-3 PUFA can greatly decrease core ASD symptoms, especially stereotypy [50,51,52,61] and social behavior [52,60] that includes social awareness [33], social motivation [57] and social-communicative functioning [33,61]. Additionally, the beneficial effects are noted in early language development [30,56] and in reducing: hyperactivity [50], repetitive behavior [52], irritability symptoms [33] and lethargy [51]. Some of the studies reported improvement of motor skills [56] as well as concentration ability [56].

However, limited support was given for the efficacy of omega-3 PUFA in reactive aggression and a short-term reduction in overall antisocial behavior [58]. No significant differences between groups in autism composite scores, adaptive function or language [55], and non-significantly greater improvement in hyperactivity [51] and in core symptoms of ASD [53] were found. No significant change was found in the score of the social interaction subscale by Doaei et al. [61].

This review has shown that high-quality studies on the effects of omega-3 PUFA supplementation in children with ASD are lacking. The available studies differ markedly in the content of supplements (EPA, DHA or both), doses and duration of supplementation, instruments used, etc. (Table 1). Moreover, in many studies, other supplements, such as vitamins or omega-6 and/or omega-9, were used. Thus, no convincing conclusion can be drawn from these data. Nevertheless, available findings on the effects of omega-3 PUFA in ASD are promising in terms of clinical efficacy and good tolerability.

## 6. Conclusions

Intestinal homeostasis, which involves a balanced status of intestinal bacteria and the gut–brain axis, prenatally as well as postnatally, seems to be one of the important factors that ameliorate the ASD clinical manifestation. Due to the wide heterogeneity across RCTs that refers to differences in methods, types and doses of PUFAs interventions (i.e., EPA or DHA or combination of the two omega-3 fatty acids or addition of omega-6 or omega-9 fatty acids), trials duration and test instruments, no definitive conclusion about omega-3 PUFAs effect can be drawn. Omega-3 PUFA effects have shown limited improvement of autism symptoms, especially on stereotyped behavior and social behavior, which includes social awareness, social motivation and communication function as well as language development, hyperactivity, repetitive behavior and motor skills and concentration in several studies. Even though up to date, no treatment is fully successful in treating ASD, a combination of complementary interventions, including PUFA interventions, which are considered very low risk, could provide added value to standard medical and psychological interventions for reducing child behavior problems. The adjuvant effects of essential fatty acid interventions are reflected in the alleviation of behavioral symptoms and could be studied as an adjunct to behavioral therapy in ASD. Supplementation with PUFAs could be considered only as one of the aspects in regulating the biological status of the organism, and the balanced biological status of the organism represents the basis for other complementary treatments to improve the psychophysiological characteristics in children with ASD. Nevertheless, in order to achieve a final conclusion on the effect of omega-3 PUFA on ASD, high-quality studies on a larger group of children with ASD and using omega-3 PUFA as the only supplementation are warranted.

## Figures and Tables

**Table 1:** RCTs of omega-3 PUFA supplementation in children with ASD.

Study	Trial Design	Drugs and Dose	Participants	Treatment Duration	Results
Amminger et al., 2007 [50]	a randomized, double-blind, placebo-controlled pilot trial	0.84 g/day EPA 0.7 g/day DHA	13 children with autistic disorders, age 5–17	6 weeks	- reduced hyperactivity and stereotypy on the ABC subscale
Bent et al., 2014 [51]	internet-based, randomized controlled trial	1.3 g of omega-3 PUFA (350 mg of EPA and 230 mg of DHA) or placebo	57 ASD children with hyperactivity (5–8 years)	6 weeks	- no improvement in hyperactivity subscale on the ABC - significant improvement in stereotypy and lethargy subscales of the ABC
Yui et al., 2012 [52]	double-blind, randomized, placebo-controlled trial	0.24 g/day DHA and 0.24 g/day AA per day	13 ASD children (6–28 years)	16 weeks	- significant improvement in social withdrawal scores on the ABC - significant increase in plasma AA; no differences in plasma DHA and EPA
Voigt et al., 2014 [53]	randomized double-blind placebo-controlled trial	DHA supplementation of 200 mg/day or placebo	48 children with ASD (3–10 years)	26 weeks	- significant positive response for the overall CGI-I rating scale - no effect on social skills on the BASC - significant improvement of functional communication on the BASC
Meguid et al., 2008 [56]	randomized, placebo-controlled trial	60 mg of DHA, 12 mg of GLA, 13 mg of EPA and 5 mg of AA	30 autistic children (3–11 years) and 30 healthy children as control	3 months	- a significant improvement in concentration ability, motor skills and language development using CARS, in twenty autistic children
Sheppard et al., 2017 [30]	pilot trial, single-site, double blinded, RCT	2.5 ml omega-3 (338 mg EPA, 225 mg DHA); 280 mg omega-6 FA(83 mg GLA); 306 mg n-9 FA	31 preterm children exhibiting ASD, 18–38 months of old born at ≤29 weeks of gestation	3 months	- significantly improved BITSEA ASD scale scores - possible improvement of early language development - significant improvement in gesture - no effect on word use alone (using CDI)
Parellada et al., 2017 [57]	a randomized, crossover, placebo-controlled study	omega-3 (EPA+DHA, 33%+22%) and vitamin E as a stabilizer Subjects 5–11 years old – EPA 577.5 mg qd + DHA 385 mg qd + vitamin E 1.6 mg qd; 12–17 years – EPA 693 mg qd + DHA 462 mg qd + vitamin E 2.01 mg qd	68 children and adolescents with ASD (5–17 years old)	8 weeks	- significant increase of omega-3/omega-6 in erythrocyte membrane - no effect on total TAS - improved all SRS scores including social motivation and communication scores - improved CGI
Raine et al., 2018 [58]	randomized, double-blind, stratified, placebo controlled, 2 × 2 factorial trial	1.12 g of omega-3 daily (411.2 mg DHA, 106.4 mg DPA, and 604 mg of EPA)	282 children with externalizing behavior disorder (7–16 years)	6 months	- significantly reduced reactive aggression and short-term reduction in overall antisocial behavior determined by RPQ, CBC, APSD, CODDS and AQ (self- or parent reported)
Mazahery et al., 2019 [33]	randomized placebo controlled double-blind study design	vitamin D (2000 IU/day, vitamin D), omega-3 LCPUFA (722 mg/day DHA, OM) or both	117 children (2.5 to 8 years) with ASD	12 months	- significant effect for SRS: improved social awareness and social communicative functioning - vitamin D reduced hyperactivity symptoms
Keim et al., 2018 [60]	randomized, fully blinded, placebo-controlled trial	omega-3-6-9 (338 mg EPA, 225 mg DHA, and 83 mg GLA) VS. canola oil (124 mg palmitic acid, 39 mg stearic acid, 513 mg linoleic acid, 225 mg *α*-linolenic acid, and 1346 mg oleic acid)	31 children born at ≤29 week of gestation (18–38 months of age)	3 months	- reduction in ASD symptoms per the BITSEA scale for the treatment group - no other scales reflected a similar magnitude or significant effect
Doaei et al., 2021 [61]	double-blind, randomized clinical trial	group (1): 1 gram omega-3 Long Chain (180 mg EPA + 120 mg DHA) and Group (2) 1 gram medium chain triglyceride as placebo	54 children with autism assigned to the case (*n* = 28) and control (*n* = 26) groups (5–15 years)	8 weeks	- significant change in autism characteristics including stereotyped behaviors and social communication at the GARS score; - no significant change in the score of social interaction subscale

**Abbreviations:** RCTs—randomized controlled trials; LCPUFA—long-chain polyunsaturated fatty acid; ASD—Autism Spectrum Disorder; EPA—eicosapentaenoic acid; DHA—docosahexaenoic acid; DPA—docosapentaenoic acid; GLA—dihomo g-linolenic acid; AA—arachidonic acid; GARS—Gilliam Autism Rating Scale; SRS—Social Responsiveness Scale; RPQ—Reactive-Proactive Aggression Questionnaire; CBC—Child Behavior Checklist; APSD—Antisocial Process Screening Device; CODDS—Conduct and Oppositional Defiant Disorder Scales; AQ—Aggression Questionnaire; BITSEA—Brief Infant Toddler Social Emotional Assessment Scale; TAS—plasma antioxidant status; CGI/ CGI-I—Clinical Global Impression-Severity/ Improvement; CDI—Mac Arthur-Bates Communicative Development Inventory; PDDBI—Pervasive Developmental Disorders Behavioral Inventory; BASC-2—Behavior Assessment; ABC—Aberrant Behavior Checklist; CARS—The Childhood Autism Rating Scale.

## Data Availability

Data sharing not applicable. No new data are created or analyzed. in this study.
